# Erratum: Recent Case Reports of Levant Blunt-Nosed Viper *Macrovipera lebetina obtusa* Snakebites in Iran

**DOI:** 10.4269/ajtmh.20-1640err

**Published:** 2022-05-18

The authors wish to correct errors in “Recent Case Reports of Levant Blunt-Nosed Viper *Macrovipera lebetina obtusa* Snakebites in Iran” by Kazemi and others (https://doi.org/10.4269/ajtmh.20-1640).

In six places in the manuscript, the authors mistakenly refer to 1-mL vials of polyvalent anti-snake venom manufactured by Razi Vaccine and Serum Research Institute in Karaj, Iran. This should have said 10-mL vials.

In addition, in the top right column on page 1871, the authors state that the patient received a dose of “1mL of polyvalent anti-snake venom which was available as lyophilized equine immunoglobulins” when this should have read “10 mL of polyvalent anti-snake venom which is derived from equine hyperimmune serum.”

The authors and the *Journal* regret these errors.

